# Application of the screening method for estimating COVID-19 vaccine effectiveness using routine surveillance data: Germany’s experience during the COVID-19 pandemic, July 2021 to March 2023

**DOI:** 10.2807/1560-7917.ES.2023.29.8.2300329

**Published:** 2024-02-22

**Authors:** Nita Perumal, Viktoria Schönfeld, Ole Wichmann

**Affiliations:** 1 Immunization Unit, Department of Infectious Disease Epidemiology, Robert Koch Institute, Berlin, Germany; 2 These authors contributed equally to this work and share first authorship

**Keywords:** COVID-19, immunisation, screening method, vaccine effectiveness, surveillance, Germany

## Abstract

The screening method represents a simple, quick, and practical tool for estimating vaccine effectiveness (VE) using routine disease surveillance and vaccine coverage data, even if these data cannot be linked. In Germany, where notification data, laboratory testing data, and vaccine coverage data cannot be linked due to strict data protection requirements, the screening method was used to assess COVID-19 VE continuously between July 2021 and March 2023. During this period, when Delta and Omicron variants circulated, VE estimates were produced in real-time for different age groups and clinical outcomes. Here we describe the country’s overall positive experience using the screening method, including its strengths and limitations, and provide practical guidance regarding a few issues, such as case definition stringency, testing behaviour, and data stratification, that require careful consideration during data analysis and the interpretation of the results.

## Background

Since the start of the worldwide COVID-19 vaccination campaign [1], it has become abundantly clear that continuous monitoring of novel COVID-19 vaccines in the real world is needed to estimate their effectiveness in the general population and specific subgroups, understand waning of vaccine-induced immunity, and assess the impact of new severe acute respiratory coronavirus 2 (SARS-CoV-2) variants on vaccine effectiveness (VE). To guide policy decision-making, such data need be available in a timely manner. Use of routine surveillance data to estimate VE represents a fast, practical, and resource-saving approach to achieve this goal. The ‘screening’ or ‘case coverage’ method (hereafter referred to as screening method) has been successfully applied to estimate effectiveness of influenza [2-5] or pneumococcal [6] vaccines by using routine disease surveillance and vaccine coverage data. While at the beginning of the pandemic it was unclear how well suited this method would be for estimating COVID-19 VE, a number of countries [7-9] successfully implemented its use early on and the World Health Organization (WHO) provided guidance for its use in June 2022 [10]. 

In Germany, real-world evidence on the effectiveness of COVID-19 vaccines was assessed using a three-pronged approach: a prospective hospital-based case–control study [11,12], the screening method using routine surveillance data, and a living systematic review to synthesise results from studies conducted worldwide [13-15]. While the other two approaches delivered reliable results with a time lag of up to a few months, the screening method was used to estimate VE continuously for different age groups and clinical outcomes with only minimal time lag during circulation of the Delta (Phylogenetic Assignment of Named Global Outbreak (Pango) lineage designation B.1.617.2) and Omicron (B.1.1.529) variants. The results were published in weekly (from July 2022 onwards, monthly) reports to inform health professionals, policy, and government decisionmakers, the media, and the public [16]. Here we describe using the screening method to estimate and report COVID-19 VE in Germany, a country where the notification data, laboratory testing data, and vaccine coverage data cannot be linked. We also discuss strengths and limitations of the method in this context.

## Screening method

The screening method estimates VE by comparing the vaccine coverage among identified cases with the vaccine coverage among the source population from which the cases arose using the following formula:


$$VE=1-\frac{PCV}{1-PCV}*\frac{1-PPV}{PPV}$$

where PCV is proportion of cases who are fully vaccinated, and PPV is the proportion of the population that is fully vaccinated [17,18]. Implementing the screening method, therefore, requires data on cases, their vaccination status, as well as on the vaccination status of the target population. In Germany, these data were collected as part of routine public health surveillance during the COVID-19 pandemic.

## German COVID-19 surveillance data

### COVID-19 case notification data

Data on cases were derived from the German national infectious disease case surveillance system (SurvNet) [19]. As with other notifiable diseases in Germany, every SARS-CoV-2 infection confirmed by culture or nucleic acid testing fulfils the COVID-19 case definition and must be notified by physicians, hospitals, laboratories, and other testing sites to local public health authorities. The local public health authorities collect information on demographics (age, sex, residence), symptoms, medical risk factors, vaccination status, and disease outcome and document this information in pseudonymised electronic case notification files, which they transmit via the federal state health authorities to the Robert Koch Institute (RKI), Germany’s national public health institute.

### COVID-19 Vaccination Coverage database

Germany began its national immunisation campaign against COVID-19 on 27 December 2020, immunising its population in order of age and risk prioritisation, with older age groups and those at higher risk of severe disease or exposure being vaccinated first [20]. Vaccine coverage data were derived from Germany’s anonymised electronic COVID-19 Vaccination Coverage database (*Digitales Impfquotenmonitoring*, DIM) [21]. The goal of DIM was to monitor in real-time the progress of Germany’s COVID-19 vaccination campaign – as there is no routine vaccination registry in Germany, providers were required to notify daily all COVID-19 vaccinations administered to the RKI, which maintains the DIM database. The Table provides a brief overview of the parameters of the screening method formula, as derived from Germany’s COVID-19 surveillance data.

**Table:** Summary and comparison of data sources, namely the COVID-19 case notification system and the COVID-19 Vaccination Coverage database, from which the parameters required for the screening method were extracted, Germany, July 2021–March 2023

Description	Vaccine coverage: proportion vaccinated in German population (PPV)	Proportion vaccinated among notified COVID-19 cases (PCV)
Data-related characteristics		
Data source	National COVID-19 Vaccination Coverage database (Digitales Impfquotenmonitoring, DIM)	Routine national infectious disease case notification system (SurvNet)
Data notification frequency	Daily	Daily
Data form	Large segments aggregated	Case-based
Variables collected		
Age	Age groups (0–4, 5–11, 12–17, 18–59, ≥ 60 years)	Age (month and year of birth)
Vaccine dose	Number of vaccine doses	Number of vaccine doses
Date	Date of last vaccine dose	Date of each vaccine dose
Case history	NA	Date of disease onset, symptoms, clinical outcome
Location	Municipality and federal state (place of vaccination)	Municipality and federal state (residence)

NA: not available.

## Calculation of COVID-19 vaccine effectiveness

COVID-19 cases were classified into vaccine ‘exposure’ categories by age group (5–11, 12–17, 18–59, and ≥ 60 years) and vaccination status; the age groups were selected to be consistent with the information collected in DIM. Unvaccinated cases were those who were notified as not having received any COVID-19 vaccine dose at disease onset. Except for children (5–11 years) for whom one COVID-19 vaccine dose was recommended (as further described), fully vaccinated individuals were defined as having received two doses of a COVID-19 vaccine ≥ 14 days before disease onset, and boosted individuals were defined as having received three or more doses of a COVID-19 vaccine ≥ 7 days before disease onset. If date of disease onset was missing, date of diagnosis or date of notification was used, in that order. Percentage of the population by the same age and vaccination categories were derived from DIM. VE for primary vaccination series (one dose for 5–11 year-olds and two doses for others) and booster doses were calculated for consecutive intervals of 4 weeks for different clinical outcomes, which were categorised according to severity of the COVID-19 course – symptomatic infection (concomitant with SARS-CoV-2 infection), hospitalisation (concomitant with SARS-CoV-2 infection), admission to an intensive care unit (ICU) (concomitant with SARS-CoV-2 infection), death (concomitant with SARS-CoV-2 infection). The VE was calculated using the standard formula provided previously and reported out from July 2021 onwards for adults (those aged ≥ 18 years) with primary doses, when prioritisation rules were lifted, and all adults became eligible for vaccination. VE for adults with booster doses was reported out from November 2021, VE for primary and booster doses among adolescents (12–17 years) were reported out from March 2022, and VE for the single primary dose for children (5–11 years) was reported out from March 2022.

## Germany’s experience

### Strengths of the screening method

Since both, COVID-19 case data and vaccine coverage data, were transmitted to the RKI daily, we were able to calculate VE estimates using the screening method with minimal extra steps and with only a minimal time lag. A lag of up to 4 weeks was usually observed for more severe clinical endpoints due to the time needed for more severe symptoms to develop, as well as due to follow-up and reporting delays. Owing to the extremely large number of reported COVID-19 cases (1.6 million symptomatic COVID-19 cases with information on vaccination status notified to SurvNet in the first 6 months of VE reporting from July to December 2021), we were able to stratify our analyses and report VE estimates for different age groups, different vaccination schemes (primary series, booster vaccinations, as well as single dose VE for 5–11 year-olds as per the national immunisation recommendations), and multiple clinical endpoints. By applying the method early in the pandemic and continuing monitoring over more than 20 months, we could follow VE over time and assess trends. 

**Figure 1:**
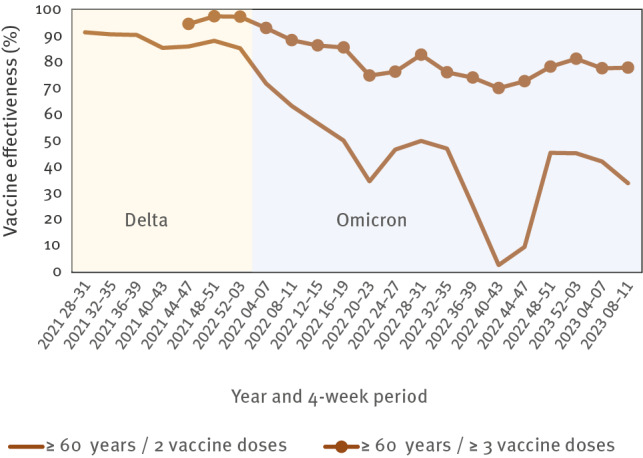
Vaccine effectiveness against hospitalisation due to COVID-19 among ≥ 60-year-olds, by number of vaccine doses, Germany, July 2021–March 2023 (in 4-week intervals)

Although information on specific variants was largely missing among case data, once a circulating variant was dominant, we attributed all new cases to that variant and interpreted the VE results accordingly. For example, after the emergence and dominance of the Omicron variant, we saw a continuous and steep decrease of VE for a two-dose scheme among adults aged ≥ 60 years, whereas VE for booster vaccinations (≥ 3 doses) exhibited a slower decrease over a period of a few weeks in this age group, thereafter stabilising and remaining high (Figure 1). The aggregated nature of the vaccine coverage data did not allow for regular assessment of VE in relation to time since vaccination (i.e. waning of vaccine-induced immunity); nonetheless, when individual vaccination histories within the target population group are available, the screening method provides a simple means of examining duration of protection. As our VE analysis sought to encompass the entire German population, with no exclusion criteria or indication of a strong selection bias, our estimates were generalisable and included even population groups that would often be excluded from clinical trials or observational studies.

### Challenges and considerations

**Figure 2:**
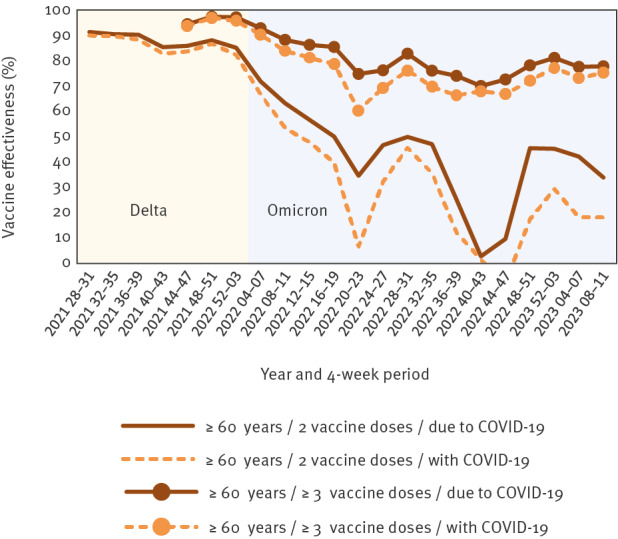
Vaccine effectiveness against hospitalisation with vs due to COVID-19 among ≥ 60-year-olds, by number of vaccine doses, Germany, July 2021–March 2023 (in 4-week intervals)

While we were able to calculate and report comprehensive, population-based VE in a timely manner, we also encountered some challenges that should be considered when applying the screening method. First, with the emergence of the Omicron variant, severity of the COVID-19 disease course decreased among both, vaccinated and unvaccinated individuals [22,23]. As a consequence, SARS-CoV-2 positive patients admitted to a hospital were often not admitted as a result of complications due to COVID-19, but increasingly for other diseases, while presenting only with mild COVID-19 symptoms. Due to routine screening for COVID-19 before all hospital admissions, however, these patients continued to be notified as hospitalised COVID-19 cases first and foremost. Using the endpoint ‘hospitalisation with COVID-19’ for our analyses led to underestimation of VE, as also seen in VE figures from England estimated using a different methodology [24]. For this reason, we retroactively narrowed the case definition for hospitalisation and ICU admission and included only cases that were ‘admitted due to COVID-19’ in our analyses, as per the recommendations of the WHO [25]. The effect of this change in case definition is shown in Figure 2. A trade-off of this decision was that it led to a decrease in the total number of cases who could be included in the VE calculations, due to a high proportion of cases without information on reason for hospitalisation.

Second, we also learned that testing behaviour was associated with vaccination status. Vaccinated individuals were more likely to test themselves (at home or in test centres) than those who were unvaccinated [26]. When mandatory testing (needed, for example, for access to schools, workplaces, restaurants, and cultural events) ended for most of the population in Summer 2022, the potential for confounding increased as vaccinated individuals continued testing themselves at a higher rate than unvaccinated individuals, leading to a sharp decline in VE for symptomatic illness. However, since routine testing for every patient at hospital admission was established and continues to this day, we were confident that this selection bias did not affect cases presenting with more severe clinical outcomes. Therefore, we decided to focus on VE against severe disease and discontinued the reporting of VE estimates for (mild) symptomatic disease.

**Figure 3:**
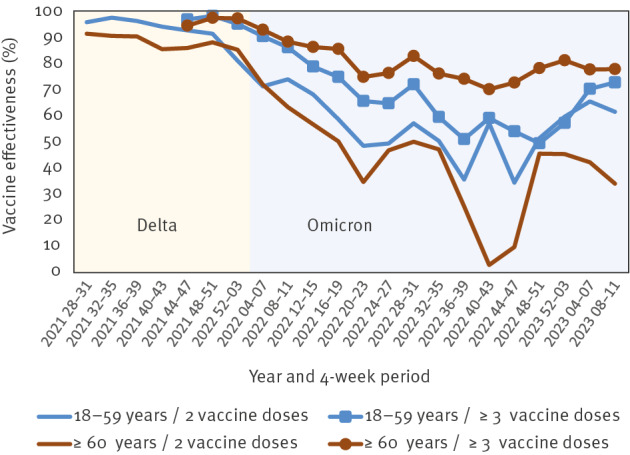
Vaccine effectiveness against hospitalisation due to COVID-19 among 18–59 and ≥ 60-year-olds, by number of vaccine doses, Germany, July 2021–March 2023 (in 4-week intervals)

Third, when using the screening method, it is imperative to adjust for confounding factors, such as by stratifying the data. As the vaccine coverage data in Germany was mostly aggregated and no or little information on comorbidities, sex, vaccine product, or narrower age groups was collected, we could only stratify our analyses for broad age groups and calendar weeks. Among the case data, information on prior infections with SARS-CoV-2 or with detail on the SARS-CoV-2 variant was largely unavailable. Thus, we observed some bias in our results and were unable to calculate VE by specific and narrower categories. Population groups at risk for severe COVID-19 were also more likely to have higher vaccine coverage. Without being able to adjust for medical risk factors, we could not rule out underestimation of VE in certain instances. A concrete example is the VE of booster doses among 18–59 year-olds in Germany, which continues to be low in general, but is likely to be higher among high risk groups within this age group. Assuming that the immune response in persons with underlying disease (especially those affecting the immune system) is lower than in healthy persons, this may have led to lower VE estimates of booster doses among younger adults in our analyses in comparison to older adults, who generally have much higher vaccine coverage due to their age itself being an indication for vaccination (Figure 3).

Fourth, we were unable to calculate reliable VE estimates among certain age groups and clinical outcome categories, such as ICU admissions or deaths among children, due to the small number of cases notified (sometimes none) for each 4-week interval. In such instances, VE fluctuated wildly over time depending on the vaccination status of the (often) one case notified for each time interval. This limitation is not specific to the screening method, however, as low sample sizes in population sub-groups is an issue faced in many study designs.

Fifth, as the data were not collected for purposes of VE estimation, a high proportion of missing values for vaccination status among hospitalised cases might have led to a non-representative sample, although we have no indication that case notification was associated with vaccination status in our data. Still, under-reporting, data incompleteness, misclassification of vaccination status or of clinical outcome, particularly during periods of high incidence, could have influenced our VE estimates.

Sixth, interpretation of results was not always simple: we regularly and retroactively compared our results with the results of other studies identified, among others, in the living systematic review [13-15], such as case–control studies with test-negative design [11,12,24], and surveillance reports published in other countries [27], in order to ensure that our screening method approach was delivering reliable results. It speaks to the overall strength of the screening method that the VE we reported out remained comparable and valid. Furthermore, it was necessary to consider the changing landscape with regards to national vaccination guidelines, emerging variants and variant-specific vaccines when analysing and interpreting the VE results for different age groups and clinical endpoints.

### Conclusions

The screening method represents a practical tool for estimating VE when surveillance data on cases and vaccine coverage among the target population are already collected, but not necessarily linked or linkable. Depending on the frequency of data collection, as well as the clinical outcome of interest, VE estimates can be calculated and reported quickly, allowing for monitoring of trends in VE over time among different population groups with only minimal time lag, as was the case in Germany during the COVID-19 pandemic. Other methods that were used to estimate VE in Germany, such as case–control studies and cohort studies, could report results with long-time lags only and required significantly greater resources; they, therefore, focused on specific clinical outcomes or population groups [11,28]. This is in contrast to countries such as England and Israel that could utilise already available national health register or health insurance data to rapidly assess population-wide VE without requiring significant resources [27,29]. Publishing the COVID-19 VE estimates, as well as the methodology of the screening method, in a transparent manner in regular reports for decisionmakers, medical experts, the media, and the public allowed for an open dialogue and showcased, through its usage, the importance of collecting reliable vaccination data. The reports also helped to demonstrate that a continuous monitoring of the COVID-19 vaccines’ effects was being conducted by national authorities and that the vaccines were and are very effective in preventing severe disease based on local, German data. Nonetheless, the screening result must be implemented within the context of its requirements and limitations and results must be interpreted with caution and used as rough guidance only.

## References

[1] World Health Organization (WHO). Evaluation of COVID-19 vaccine effectiveness: Interim guidance (17 March 2021). Geneva: WHO; 2021. Available from: https://www.who.int/publications/i/item/WHO-2019-nCoV-vaccine_effectiveness-measurement-2021.1

[2] Thomas HL Andrews N Green HK Boddington NL Zhao H Reynolds A Estimating vaccine effectiveness against severe influenza in England and Scotland 2011/2012: applying the screening method to data from intensive care surveillance systems. Epidemiol Infect. 2014;142(1):126-33. 10.1017/S0950268813000824 23591102 PMC9152609

[3] Legrand J Vergu E Flahault A. Real-time monitoring of the influenza vaccine field effectiveness. Vaccine. 2006;24(44-46):6605-11. 10.1016/j.vaccine.2006.05.063 16806607

[4] Remschmidt C Rieck T Bödeker B Wichmann O. Application of the screening method to monitor influenza vaccine effectiveness among the elderly in Germany. BMC Infect Dis. 2015;15(1):137. 10.1186/s12879-015-0882-3 25887460 PMC4371628

[5] Seyler T Bella A Puzelli S Donatelli I Rizzo C screening method working group. Estimating pandemic vaccine effectiveness in two Italian regions using the screening method, 2009-2010. Vaccine. 2012;30(2):109-11. 10.1016/j.vaccine.2011.11.013 22100893

[6] Cohen AL Taylor T Jr Farley MM Schaffner W Lesher LJ Gershman KA An assessment of the screening method to evaluate vaccine effectiveness: the case of 7-valent pneumococcal conjugate vaccine in the United States. PLoS One. 2012;7(8):e41785. 10.1371/journal.pone.0041785 22870248 PMC3411566

[7] Moline HL Whitaker M Deng L Rhodes JC Milucky J Pham H Effectiveness of COVID-19 Vaccines in Preventing Hospitalization Among Adults Aged ≥65 Years - COVID-NET, 13 States, February-April 2021. MMWR Morb Mortal Wkly Rep. 2021;70(32):1088-93. 10.15585/mmwr.mm7032e3 34383730 PMC8360274

[8] Horváth JK Ferenci T Ferenczi A Túri G Röst G Oroszi B. Real-Time Monitoring of the Effectiveness of Six COVID-19 Vaccines against Laboratory-Confirmed COVID-19 in Hungary in 2021 Using the Screening Method. Vaccines (Basel). 2022;10(11):1824. 10.3390/vaccines10111824 36366334 PMC9697606

[9] Mazagatos C Monge S Olmedo C Vega L Gallego P Martín-Merino E Working Group for the surveillance and control of COVID-19 in Spain Working group for the surveillance and control of COVID-19 in Spain. Effectiveness of mRNA COVID-19 vaccines in preventing SARS-CoV-2 infections and COVID-19 hospitalisations and deaths in elderly long-term care facility residents, Spain, weeks 53 2020 to 13 2021. Euro Surveill. 2021;26(24):2100452. 10.2807/1560-7917.ES.2021.26.24.2100452 34142647 PMC8212595

[10] World Health Organization (WHO). COVID-19 vaccine effectiveness estimation using the screening method: Operational tool for countries. Geneva: WHO. 2022.

[11] Stoliaroff-Pepin A Peine C Herath T Lachmann J Hellenbrand W Perriat D Vaccine effectiveness against severe COVID-19 during the Omicron wave in Germany: results from the COViK study. Infection. 2023;51(4):1093-102. 10.1007/s15010-023-02012-z 36913112 PMC10009838

[12] Stoliaroff-Pepin A Peine C Herath T Lachmann J Perriat D Dörre A Effectiveness of vaccines in preventing hospitalization due to COVID-19: A multicenter hospital-based case-control study, Germany, June 2021 to January 2022. Vaccine. 2023;41(2):290-3. 10.1016/j.vaccine.2022.11.065 36509640 PMC9715487

[13] Harder T Koch J Vygen-Bonnet S Külper-Schiek W Pilic A Reda S Efficacy and effectiveness of COVID-19 vaccines against SARS-CoV-2 infection: interim results of a living systematic review, 1 January to 14 May 2021. Euro Surveill. 2021;26(28):2100563. 10.2807/1560-7917.ES.2021.26.28.2100563 34269175 PMC8284046

[14] Harder T Külper-Schiek W Reda S Treskova-Schwarzbach M Koch J Vygen-Bonnet S Effectiveness of COVID-19 vaccines against SARS-CoV-2 infection with the Delta (B.1.617.2) variant: second interim results of a living systematic review and meta-analysis, 1 January to 25 August 2021. Euro Surveill. 2021;26(41):2100920. 10.2807/1560-7917.ES.2021.26.41.2100920 34651577 PMC8518304

[15] Külper-Schiek W Piechotta V Pilic A Batke M Dreveton LS Geurts B Facing the Omicron variant-how well do vaccines protect against mild and severe COVID-19? Third interim analysis of a living systematic review. Front Immunol. 2022;13:940562. 10.3389/fimmu.2022.940562 36091023 PMC9449804

[16] Robert Koch Institute (RKI). Monitoring des COVID-19-Impfgeschehens in Deutschland: Monatsbericht [Monthly monitoring report on COVID-19 vaccination in Germany]. Edited by Department of Infectious Disease Epidemiology. Berlin: RKI. 2022-2023.

[17] Farrington CP. Estimation of vaccine effectiveness using the screening method. Int J Epidemiol. 1993;22(4):742-6. 10.1093/ije/22.4.742 8225751

[18] Mary-Krause M Mary JY Valleron AJ. Conditions of validation and use of the screening method for vaccine efficacy evaluation. Rev Epidemiol Sante Publique. 1993;41(2):155-60. 8493394

[19] Faensen D Claus H Benzler J Ammon A Pfoch T Breuer T SurvNet@RKI--a multistate electronic reporting system for communicable diseases. Euro Surveill. 2006;11(4):614. 10.2807/esm.11.04.00614-en 16645245

[20] Perumal N Steffen A Ullrich A Siedler A. Impact of COVID-19 immunisation on COVID-19 incidence, hospitalisations, and deaths by age group in Germany from December 2020 to October 2021. Vaccine. 2022;40(21):2910-4. 10.1016/j.vaccine.2022.04.002 35428498 PMC8990687

[21] Robert Koch Institute. Digitales Impfquotenmonitoring zur COVID-19-Impfung [COVID-19 vaccination coverage surveillance in Germany]. Available at www.rki.de/covid-19-impfquoten.

[22] Sievers C Zacher B Ullrich A Huska M Fuchs S Buda S SARS-CoV-2 Omicron variants BA.1 and BA.2 both show similarly reduced disease severity of COVID-19 compared to Delta, Germany, 2021 to 2022. Euro Surveill. 2022;27(22):2200396. 10.2807/1560-7917.ES.2022.27.22.2200396 35656831 PMC9164675

[23] Relan P Motaze NV Kothari K Askie L Le Polain de Waroux O Van Kerkhove MD Severity and outcomes of Omicron variant of SARS-CoV-2 compared to Delta variant and severity of Omicron sublineages: a systematic review and metanalysis. BMJ Glob Health. 2023;8(7):e012328. 10.1136/bmjgh-2023-012328 37419502 PMC10347449

[24] Stowe J Andrews N Kirsebom F Ramsay M Bernal JL. Effectiveness of COVID-19 vaccines against Omicron and Delta hospitalisation, a test negative case-control study. Nat Commun. 2022;13(1):5736. 10.1038/s41467-022-33378-7 36180428 PMC9523190

[25] World Health Organization (WHO). Evaluation of COVID-19 vaccine effectiveness in a changing landscape of COVID-19 epidemiology and vaccination (Second addendum to Evaluation of COVID-19 vaccine effectiveness: Interim guidance). Geneva: WHO. 2022.

[26] Betsch C, Eitze S, Sprengholz P, Korn L, Shamsrizi P, Geiger M, et al. Ergebnisse aus dem COVID-19 Snapshot MOnitoring COSMO. Stand 02.12.22. [Results from the COVID-19 Snapshot Monitoring (COSMO) study: Update from December 2022]; Dec 2022. German. Available from: https://projekte.uni-erfurt.de/cosmo2020/files/COSMO_W70.pdf

[27] UK Health Security Agency (UKHSA). COVID-19 vaccine quarterly surveillance reports (September 2021 to October 2023). London: UKHSA. 2023.

[28] Meyer ED Sandfort M Bender J Matysiak-Klose D Dörre A Bojara G BNT162b2 vaccination reduced infections and transmission in a COVID-19 outbreak in a nursing home in Germany, 2021. Influenza Other Respir Viruses. 2023;17(1):e13051. 10.1111/irv.13051 36082799 PMC9538000

[29] Barda N Dagan N Cohen C Hernán MA Lipsitch M Kohane IS Effectiveness of a third dose of the BNT162b2 mRNA COVID-19 vaccine for preventing severe outcomes in Israel: an observational study. Lancet. 2021;398(10316):2093-100. 10.1016/S0140-6736(21)02249-2 34756184 PMC8555967

